# Association between Different Modes of Travelling and Adiposity in Chilean Population: Findings from the Chilean National Health Survey 2016–2017

**DOI:** 10.3390/ijerph17103731

**Published:** 2020-05-25

**Authors:** Ignacio Medina, Fanny Petermann-Rocha, Heather Waddell, Ximena Díaz-Martínez, Carlos Matus-Castillo, Igor Cigarroa, Yeny Concha-Cisternas, Carlos Salas-Bravo, Maria A Martínez-Sanguinetti, Carlos Celis-Morales, ELHOC-Research Team

**Affiliations:** 1 British Heart Foundation Glasgow Cardiovascular Research Centre, Institute of Cardiovascular and Medical Sciences, University of Glasgow, Glasgow G12 8TA, UK; imedinamarchant@gmail.com (I.M.); f.petermann-rocha.1@research.gla.ac.uk (F.P.-R.); h.waddell@sms.ed.ac.uk (H.W.); 2 Institute of Health and Wellbeing, University of Glasgow, Glasgow G12 8RZ, UK; 3 Medical Research Council Centre for Inflammation Research, The Queen’s Medical Research Institute, The University of Edinburgh, Edinburgh EH16 4TJ, UK; 4 Grupo de Investigación en Calidad de Vida, Departamento de Ciencias de la Educación, Facultad de Educación y Humanidades, Universidad del Biobío, Chillán 378000, Chile; 5 Departamento de Ciencias del Deporte y Acondicionamiento Físico, Universidad Católica de la Santísima Concepción, Concepción 4090541, Chile; cmatus@ucsc.cl; 6 Escuela de Kinesiología, Facultad de Salud, Universidad Santo Tomás, Santiago 8370003, Chile; icigarroa@santotomas.cl (I.C.); yenyf.concha@gmail.com (Y.C.-C.); 7 Pedagogía en Educación Física, Facultad de Educación, Universidad Autónoma de Chile, Talca 3467987, Chile; 8 Departamento de Educación Física, Facultad de Educación, Universidad de Concepción, Concepción 4070386, Chile; 9 Instituto de Farmacia, Facultad de Ciencias, Universidad Austral de Chile, Valdivia 5090000, Chile; mmartin3@uach.cl; 10 Centro de Investigación en Fisiología del Ejercicio (CIFE), Universidad Mayor, Santiago 7510041, Chile; 11 Laboratorio de Rendimiento Humano, Grupo de Estudio en Educación, Actividad Física y Salud (GEEAFyS), Universidad Católica del Maule, Talca 3480112, Chile

**Keywords:** active travel, physical activity, adiposity, transportation, obesity

## Abstract

*Background:* Active travel has been suggested as a feasible way of increasing physical activity levels. Although international studies have demonstrated its effect over different health outcomes and adiposity, there is still limited evidence on this topic in developing countries, such as Chile. *Aim:* To investigate the associations between different types of travelling and markers of obesity in the Chilean adult population. *Methods:* 5411 participants from the Chilean National Health Survey 2016–2017 (CNHS) were included in this study. Active travel was assessed using a questionnaire. Car commuters, public transport (PT), walking and cycling were the four forms of travelling assessed. Bodyweight, body mass index and waist circumference were used as markers of adiposity. *Results:* Compared to car travellers, body weight, WC and BMI levels were lower for PT walking and cycling travellers. The odds for obesity (Odds ratio (OR): 0.41 (95% CI: 0.28; 0.61 *p* ≤ 0.001) were lower for walking and the odds (OR: 0.56 (95%CI: 0.35; 0.89 *p* = 0.014) for central obesity were significantly lower for cyclist in comparison to car travellers. Additionally, participation in any form of active travel (walking or cycling) was low, with only 20.9% of the population reporting being active travellers. *Conclusion:* Active travel, such as walking and cycling, was associated with lower adiposity levels in the Chilean adult population. Promoting active travel could be a feasible strategy to tackle the high prevalence of obesity and physical inactivity in the Chilean population.

## 1. Introduction

It has been widely proven that high physical activity (PA) levels are associated with several health benefits, including lower risk for type 2 diabetes, hypertension, cardiovascular disease, some types of cancer and premature mortality [1,2,3,4,5]. Furthermore, physical inactivity is responsible for more than five million deaths globally, and is currently cited as the fourth leading cause of non-communicable diseases [6,7]. Despite this knowledge about the benefits of increasing PA levels, the prevalence of physical inactivity continues to rise each year with nearly one-third of the population not meeting the current PA guidelines [8].

Urbanisation and industrialisation have been reported as the main factors associated with decreased PA levels [9,10,11]. Although several strategies have been implemented to increase the PA of the population [12], a high proportion remain physically inactive [8]. Active travel, such as walking or cycling, has been suggested as a feasible approach to increasing PA and reducing the burden of non-communicable diseases [13,14]. To date, several studies have reported that active travel and active commuting (travelling to and from work) are associated with better health outcomes, including lower risk of obesity, cardiovascular diseases, cancer and premature mortality. Moreover, in active commuting, a recent cycling commuting trial conducted in overweight and obese individuals found that a six-month bike-commuting intervention was associated with improved insulin sensitivity, cardiorespiratory fitness and lower abdominal adipose tissue [15]. Interestingly, the health improvements observed of those randomised to the bike-commuting group were similar to those individuals allocated to the vigorous-intensity PA intervention [15]. Likewise, a recent systematic review identified a positive relationship between active commuting and physical fitness levels in adults [16].

Although evidence regarding the health benefits of active travel is increasing, most of these studies have been conducted in high-income countries [3,17,18,19,20,21,22,23], with limited evidence available for middle- and low-income regions, like Latin America [24,25,26,27,28]. Chile has experienced a fast economic growth and lifestyle transition in the last three decades, compared to other Latin American countries [29,30,31]. These lifestyles changes have caused the Chilean population to become ranked first, across all Latin America countries, for having the highest levels of obesity and cardiovascular risk factors [32,33]. To date, more than 70% of the Chilean population is overweight or obese, and 26.6% is physically inactive [8,34,35]. This scenario is unlikely to change if the current levels of inactivity of the population are not reduced [36]. Active travel could be a feasible approach to increase the PA levels of the population and address the current health profile in Chile [24,27]. However, there is limited evidence on the association of types of transport used with adiposity and obesity levels in Chile [24,26,28,37,38]. The aim of this study, therefore, was to investigate the associations between different modes of travelling and adiposity in the Chilean adult population.

## 2. Materials and Methods

### 2.1. Study Design

This cross-sectional study included 5411 participants, aged between 18 and 96 years old, from the 2016–2017 Chilean National Health Survey who have full data available for types of transport adiposity outcomes and covariates [39]. All participants provided written consent prior to participation.

The CNHS is a nationally representative health household survey with a stratified multistage probability sample of 6233 non-institutionalised participants aged ≥15 years from urban and rural Chile, including the 15 Chilean geographical regions [39]. The data collection was performed between August 2016 and March 2017. One participant per household was randomly selected using a computational Kish algorithm. Response rate was 67%; refusal rate was 9.8%, with no replacements. The CNHS 2016–2017 was funded by the Chilean Ministry of Health and approved by the Ethics Research Committee of the School of Medicine at the Pontificia Universidad Católica de Chile (No. 16–019) [39].

### 2.2. Anthropometric Measurements

Height was measured using a portable stadiometer, which is accurate to the nearest 0.1 cm, in conjunction with weight, measured using a digital scale (Tanita HD313) to the nearest 0.1 kg. Weight measurements were taken in bare feet and participants wore light items of clothing [39]. Body mass index (BMI) was calculated by weight/height^2^ and categorized using the World Health Organization criteria (Underweight ≤18.5 kg/m^2^, Normal 18.5–24.9 kg/m^2^, overweight 25.0–29.9 kg/m^2^ and obesity ≥30.0 kg/m^2^) [39]. Central obesity was determined by measuring waist circumference (WC). This was further split by sex, in accordance with the standardised protocol; central obesity for female participants was defined as >88 cm and >102 cm for male participants [39].

### 2.3. Physical Activity and Types of Travelling

To measure PA, the Global Physical Activity Questionnaire (GPAQ v2) was used [40]. This questionnaire uses standardised protocols proven to be a valid, reliable and adaptable method to assess differences among various populations with cultural and behavioural differences. To assess active travel, participants were asked the following questions: (i) Do you walk or cycle for at least 10 min continuously to get to and from places? (Yes, No); (ii) in a typical week, on how many days do you walk or cycle for at least 10 min continuously? (iii) how much time do you spend walking or cycling for travelling on a typical day? These questions were used to derive time spent on active travel in minutes per day [40]. The four modes of travel considered for this study were collected using a questionnaire and included cars, public transport (PT), walking and cycling. Sedentary behaviour (time spent sitting, reclining or lying down) was measured using the same questionnaire whereby the question asked was: ‘How much time do you usually spend sitting or reclining on a typical day?’ [40].

### 2.4. Socio-Demographic and Lifestyle

Socio-demographic and lifestyle data were collected from all participants using previously validated questionnaires utilised in the CNHS 2016–2017 [40]. Age, sex, education level (primary, secondary or beyond secondary) and place of residency (urban or rural) were self-reported. Lifestyle factors, such as daily fruit and vegetable intake and smoking (non-smoker, current smoker, smoker, occasional smoker) were self-reported [40]. Alcohol consumption was also self-reported and collected using the “Alcohol Use Disorders Identification Test” (AUDIT) questionnaire, developed by the World Health Organization [41]. The “units of alcohol” indicator represented the number of self-reported 200 mL glasses of alcohol consumed. This was a standard measure adapted from one of the ten items of the AUDIT questionnaire, and an AUDIT score >8 was used as a cut-off point for hazardous consumption [41].

### 2.5. Statistical Analyses

All statistical analyses were conducted using STATA 15 software (StataCorp; College Station, TX). Analyses were weighted by the survey design [39]. A *p*-value < 0.05 was set as statistically significant.

The descriptive characteristics of the cohort are presented as means or proportion with their 95% confidence intervals (95% CI) for continuous, and categorical variables, respectively. In order to investigate the associations between the different types of travel modes and adiposity levels, we used a linear regression model to obtain the beta coefficients and their 95% CI. Additionally, logistic regression analyses were performed with the results presented as odds ratios (OR) and 95% confidence intervals (CI). In the statistical models, car travellers were used as the reference group.

All statistical analyses were incrementally adjusted by several confounding factors including four models: Model 1 was unadjusted; Model 2 was adjusted by socio-demographic factors (age, sex, education and place of residency), Model 3 was additionally adjusted by lifestyle factors (smoking behaviour, alcohol consumption, fruit and vegetable intake and sedentary behaviours) and finally, Model 4 was further adjusted by levels of leisure PA.

## 3. Results

Descriptive characteristics by types of transport are presented in Table 1. In summary, more than half of the population used PT as their main form of transportation (52.4%). The prevalence of those using PT was greater in women (61.3%) compared to men. On the other hand, men reported travelling by car as their main form of transportation (66.4%). Active travel was achieved only by 20.9% of the population (6.3% and 14.6% of cycling, and walking, respectively). From 6.3% of the population that cycle, 75.9% were men.

Compared to cars and walking, those who reported cycling or using PT as their main travelling modes were younger than those who did not. Travelling by car was also more frequently reported by those who had a university or college degree, whereas PT, cycling and walking were more common in individuals with primary and secondary education. Additionally, the lowest prevalence of obesity was observed in those who cycle, followed by walking, whereas the highest was identified for car and PT (39.2% versus 28.5%). Similarly, those who cycle reported the highest levels of total and vigorous PA with an average of 1588.1(MET/min/day) and 255.1(min/day), respectively. Physical inactivity levels were higher in car and PT travellers (~28%). However, it was considerably lower in cycling and walking travellers (7.7% and 14.7%, respectively). On the other hand, sedentary behaviours were also lower in cycling and walking travellers, compared to car and PT travellers (Table 1).

**Figure 1:**
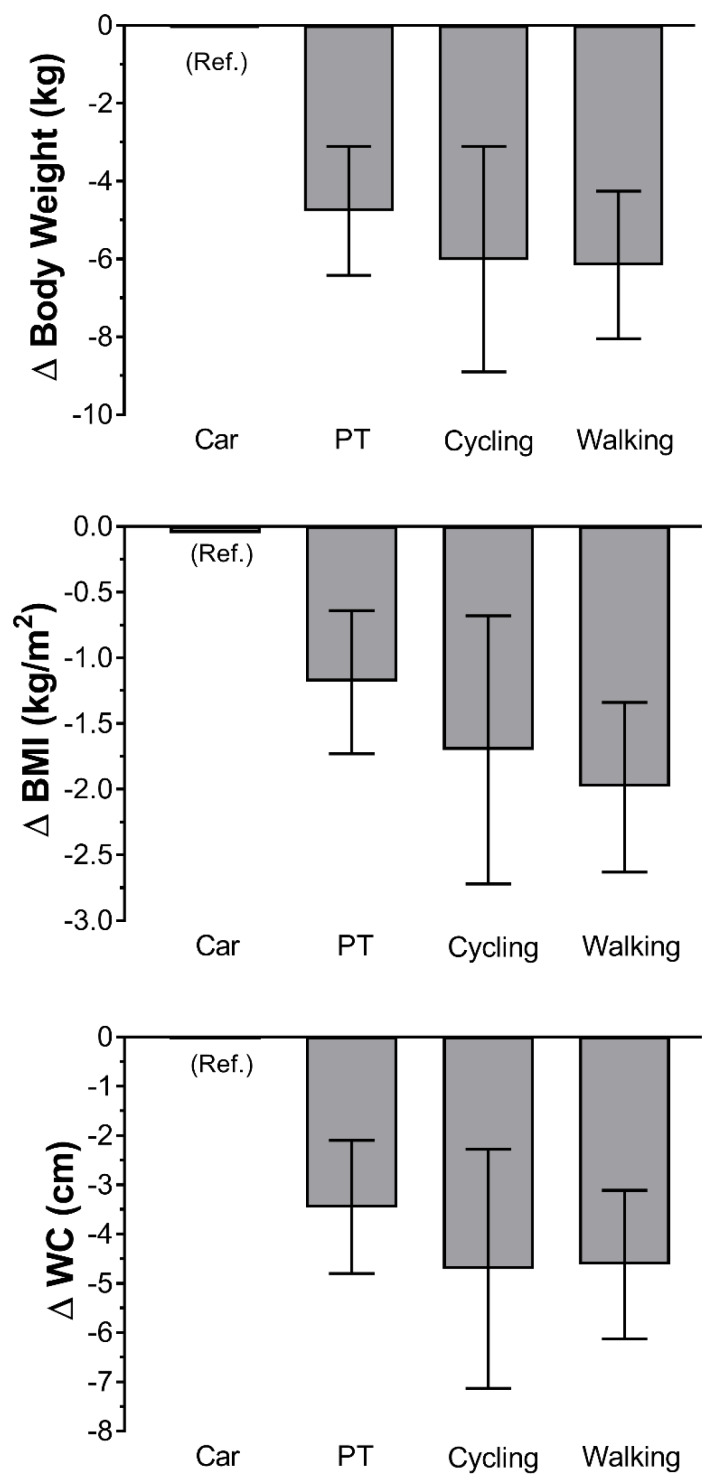
Association between travel mode and adiposity markers. Data presented as β coefficient and their 95% confidence intervals. Car travellers were used as the reference group; therefore, the β indicate differences of each travel mode compared to cars travellers. Analyses were adjusted by socio-demographic factors (age, sex, education and place of residency), lifestyle factors (smoking, alcohol, fruit and vegetable intake and sedentary behaviours) and leisure PA. BMI: body mass index; WC: waist circumference; β: beta coefficient.

Table 2 describes the association between the different modes of travel and adiposity markers (body weight, BMI and WC). Overall, compared to car travellers, PT, cycling and walking travellers had lower body weight, BMI and WC (Table 2). For the most adjusted model (Model 4) those who walk had −6.16 kg lower body weight, −1.98 kg/m^2^ lower BMI and −4.62 cm lower WC than car travellers. Similarly, cyclist had a −6.03 kg lower bodyweight, −1.70 kg/m^2^ lower BMI and −4.71 cm lower WC. Slight lower differences were observed for PT versus car travellers (−4.77 kg lower bodyweight, −1.18 kg/m^2^ lower BMI and −3.46 cm lower WC) (Table 2 and Figure 1).

**Figure 2:**
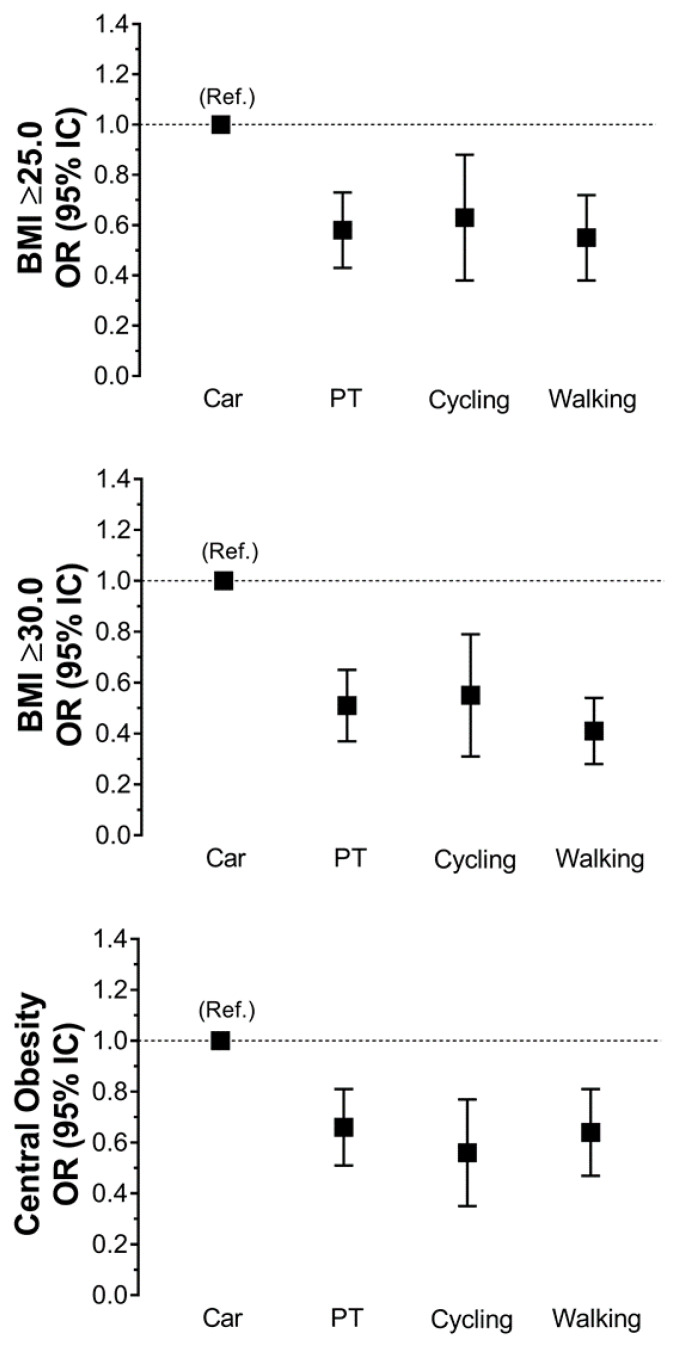
Association between travel mode and obesity outcomes. Data presented as odds ratio and their 95% confidence intervals. Car travellers were used as a reference. Analyses were adjusted by socio-demographic factors (age, sex, education and place of residency), lifestyle factors (smoking, alcohol, fruit and vegetable intake and sedentary behaviours) and leisure PA. WC: waist circumference; β: beta coefficient. Central obesity was defined as WC >88 and >102 cm for men and women, respectively.

The associations of travelling mode with overweight, obese and central obesity are presented in Table 3. Overall, PT, cycling and walking travellers had a lower odd of overweight, obesity and central obesity compared to car travellers. The odds ratio for obesity for the most adjusted model (Model 4) were 0.55 [95% CI: 0.31; 0.99] for cycling, 0.41 [95% CI: 0.28; 0.61] for walking and 0.51 [95% CI: 0.37; 0.72] for PT, compared to car travellers. Similar associations were observed for overweight and central obesity (Table 3 and Figure 2).

## 4. Discussion

### 4.1. Main Findings of This Study

Active travel was associated with lower adiposity levels (body weight, BMI and WC), with cycling and walking travellers presenting the lowest adiposity levels. Furthermore, PT, cycling, and walking as part of the commute were also associated with lower odds of obesity. Our findings reveal that the associations observed between travelling mode and obesity-related outcomes were independent of major confounding factors, including age, sex, education, diet, time spent sitting and leisure PA. Engaging the population in regular PA has proved to be challenging. Therefore, active travel (including active commuting) may offer a feasible alternative for increasing PA in the population. This could result in important health-related benefits, including reduced obesity and potentially obesity-related illnesses.

Chile is an emerging economy, and as part of recent developments, the population has adopted a swesternised lifestyle, characterised by poor diet and lack of PA, which has led to an increasing prevalence in obesity and non-communicable diseases [29,30,31,33]. Therefore, cycling or walking, including to, and from, work could offer important health benefits in the Chilean population. However, only 20.9% of the population reported performing any form of active travel, and from this, only 6.3% reported cycling as their most common mode of travel. This finding suggests that there might be several barriers to active travelling in Chile, such as lack of infrastructure, safety regulations and policies that discourage active travel [42].

### 4.2. What is Already Known in This Topic?

Interestingly, PT travellers showed lower adiposity levels compared with car travellers. A similar study was conducted on 156,666 British adults from the UK Biobank, investigating modes of travelling. The study found that participants who reported PT as their main mode of travelling, on average, has a lower BMI of −0.70 kg/m^2^ than car commuters. Differences in BMI between car and PT commuters reported in the UK Biobank population were lower than the observed in our study (−0.70 vs −1.18 kg/m^2^). This may be explained by mixed-mode travelling, as UK Biobank participants who reported using PT but also cycling and walking had a −1.00 kg/m^2^ lower BMI than car commuters [43]. Our findings also corroborate previous evidence reporting that cycling or walking as part of the commuting is associated with lower levels of body weight, BMI and WC [15,17,18,19,26]. A study conducted in UK Biobank participants reported that compared to cars travellers, BMI levels were lower for those who cycle (−1.71 kg/m^2^) or walk (−0.98 kg/m^2^) as part of their travel. Although our study reported similar differences in BMI between those who use cars versus cycling (−1.70 kg/m^2^), the difference for those who walk (−1.98 kg/m^2^) was larger than the reported in the UK Biobank study [43]. We also found that cyclists had the lowest prevalence of abdominal obesity (29.1%) and physical inactivity (7.7%), as well as the highest levels of physical activity (1588 MET-min/day) across all types of transportation. These findings are in agreement with a large study conducted in 263,450 British participants, where cyclists had the lowest prevalence of central obesity (13.4%) and physical inactivity (9.8%). However, the cyclists still reported the highest levels of physical activity (643.9 MET-min/day) compared with other types of transport (car, PT or walking) [3].

Although there is a large amount of evidence on commuting and health, generated from high-income countries [3,17,18,19,20,21,22,23], evidence from low- or middle-income countries, especially from Latin America and Chile, is limited [24,25,26,27,28,44]. A recent study conducted in Chilean adults reported that per every 30 min spent actively commuting (cycling or walking) the odds for obesity, diabetes and metabolic syndrome was 10%, 19% and 14% lower [26]. Similarly, compared to those who reported not doing any form of active commuting, those who actively commute for at least >30 min per day have lower levels of BMI, WC, fasting glucose and systolic blood pressure [26]. Another study conducted in Colombian university students reported similar associations, where those who engaged in a walking commuting had a 55% lower odds for obesity, 74% lower odds for high blood pressure and 71% lower odds for low HDL cholesterol compared to non-active commuters [45].

### 4.3. What This Study Adds

Despite the low prevalence of active travel amongst the population, active travellers (walking and cycling) showed lower levels of body weight, BMI and WC but also lower odds of obesity. These findings suggest that promoting active travel may lead to lower obesity prevalence in Chile. As mentioned earlier in this manuscript, Chile has the highest levels of obesity and cardiovascular risk factors amongst Latin American countries [32,33]. Therefore, novel strategies, such as promoting active travel (including active commuting) could be implemented due to the fact that this form of PA would be part of people’s daily routine, which may be more likely to be successfully adopted than PA programs based on leisure PA [46]. Additionally, our findings suggest that the low prevalence of active travel in Chile may be related to a lack of appropriate infrastructure, poor safety perception from the population and a lack of relevant legislation on road safety for cyclists and active commuters [42]. Other barriers, such as lack of time and lack of motivation have been cited as common barriers to people engaging in regular PA [11].

### 4.4. Limitations of This Study

The cross-sectional nature of this type of study limited our ability to assess causality. Therefore, the findings reported should be interpreted with caution. Despite the adjustment for several confounding factors, we cannot rule out the effect of other key covariates that were not measured in the Chilean Health survey, such as pollution, safety, distance from train or bus stops and dietary intake, which could explain, in part, some of the association found between travelling and adiposity in our study. Additionally, the self-reported nature of travelling data may be prone to misreporting bias, which could obscure the true nature and magnitude of associations found. However, there is no gold standard for measuring commuting modes at the population level. Although we were able to identify different travelling mode, we were not able to distinguish between active travel and active commuting to, and from, work. Finally, the survey did not include information on whether the individuals use more than one form of travel. Therefore, the current results are only based on the most common type of transport used.

## 5. Conclusions

In conclusion, the present study corroborates that active and PT travelling mode were associated with lower adiposity levels and lower odds for presenting obesity in the Chilean population. Therefore, public health institutions should strongly consider implementing active travel policies in their intervention strategies to encourage people to become more active, which in turn, could help to decrease the alarming levels of physical inactivity and the rise in the obesity and non-communicable diseases in the Chilean population.

## Figures and Tables

**Table 1:** Descriptive characteristics of participants by active travel categories.

	Cars	Public Transport	Cycling	Walking
%	26.7 (24.7; 28.8)	52.4 (50.0; 54.8)	6.3 (5.2; 7.6)	14.6 (13.3; 16.2)
**Sociodemographics**				
Age (years)	44.4 (43.1; 45.7)	42.4 (41.2; 43.5)	41.7 (38.8; 44.6)	43.8 (41.4; 46.1)
**Age categories, %**				
Younger (<37 years)	41.1 (36.7; 45.7)	46.8 (43.4; 50.2)	46.9 (37.0; 57.1)	44.2 (38.8; 49.7)
Middle age (37–56 years)	39.8 (35.5; 44.3)	31.8 (28.7; 35.2)	38.3 (29.5; 47.9)	31.2 (26.4; 36.6)
Older adults (>56 years)	19.1 (16.1; 22.6)	21.4 (19.1; 23.8)	14.7 (9.9; 21.4)	24.6 (20.4; 29.3)
**Sex, %**				
Women	33.6 (29.8; 37.5)	61.3 (57.8; 64.6)	24.1 (16.7; 33.5)	58.4(52.7; 63.8)
Men	66.4 (62.4; 70.2)	38.7 (35.4; 42.2)	75.9 (66.4; 83.3)	41.6 (36.1; 47.3)
**Education**				
Primary	12.4(9.8; 15.5)	16.2 (14.2; 18.4)	16.7 (11.0; 24.5)	24.1 (19.8; 29.0)
Secondary	52.1 (47.5; 56.6)	56.3; (52.9; 59.7)	59.7 (49.4; 69.3)	59.4 (53.8; 64.8)
University/technical degree	35.5 (31.2; 40.1)	27.5 (24.4; 30.8)	23.6 (15.2; 34.7)	16.5 (12.4; 21.6)
**Place of residency, %**				
Urban	87.1 (84.5; 89.3)	91.2 (89.8; 92.4)	87.9 (82.1; 90.1)	84.6 (80.8; 87.7)
Rural	12.8 (10.6; 15.4)	8.7 (7.5; 10.2)	12.0 (7.9; 17.8)	15.3 (12.2; 19.1)
**Anthropometrics**				
Height (m)	1.66 (1.65; 1.67)	1.61 (1.60; 1.62)	1.66 (1.64; 1.68)	1.61 (1.60; 1.62)
Weight (kg)	80.9 (79.7; 82.2)	73.6 (72.5; 74.7)	75.6 (72.9; 78.2)	72.4 (70.9; 73.8)
BMI (kg/m^2^)	29.4 (28.9; 29.8)	28.4 (28.0; 28.8)	27.5 (26.4; 28.5)	27.8 (27.3; 28.3)
**Nutritional status, %**				
Underweight	0.1 (0.0; 0.3)	1.5 (0.9; 2.6)	3.8 (1.2; 11.1)	1.4 (0.6; 3.1)
Normal weight	17.6 (14.5; 21.2)	27.1 (24.2; 30.3)	25.0 (17.0; 35.1)	27.4 (22.9; 32.5)
Overweight	43.1 (38.7; 47.7)	37.4 (34.1; 40.8)	42.7 (33.2; 52.8)	41.4 (36.1; 47.1)
Obese	39.2 (34.9; 43.6)	33.9 (30.8; 37.2)	28.5 (20.7; 37.8)	29.7 (25.1; 34.8)
WC (cm)	96.6 (95.8; 97.8)	92.1 (91.1; 93.1)	91.8 (89.3; 94.3)	91.4 (90.2; 92.7)
**Central obesity, %**				
Normal	55.6 (51.1; 59.9)	55.0 (51.6; 58.3)	70.9 (61.7; 78.7)	54.7 (49.2; 60.0)
Obese	44.4 (40.1; 48.9)	45.0 (41.7; 48.4)	29.1 (21.3; 38.3)	45.3 (40.0; 50.8)
**Lifestyle**				
Physical inactivity, %	28.6 (24.8; 32.7)	28.4 (25.6; 31.4)	7.7(3.8; 14.8)	14.7(11.4; 18.8)
Transport PA (min.day^−1^)	64.3 (526; 76.1)	67.4 (57.7; 77.0)	78.8 (63.1; 94.4)	84.0 (72.6; 95.4)
Moderate PA (min.day^−1^)	236.2 (196.8; 275.7)	226.4 (206.0; 246.9)	240.7 (187.1; 294.4)	206.7 (177.8; 235.7)
Vigorous PA (min.day^−1^)	216.0 (183.3; 248.8)	213.1 (186.6; 239.6)	255.1 (189.0; 301.2)	182.9 (150.0; 215.8)
Total PA (MET-min.day^−1^)	1274.7 (1088; 1461)	1097.4 (994; 1200)	1588.1 (1278; 1898)	1132.4 (996; 1268)
Sitting (min.day^−1^)	209.7 (193.4; 226.0)	215.5 (203.4; 227.7)	161.6 (131.0; 192.3)	169.5 (150.0; 189.0)
**Fruit and vegetables, %**				
<5 portions.day^−1^	0.87 (0.84; 0.90)	0.85 (0.82; 0.87)	0.81 (0.70; 0.88)	0.84 (0.79; 0.88)
≥5 portions.day^−1^	0.13 (0.10; 0.16)	0.15 (0.13; 0.18)	0.19 (0.12; 0.30)	0.16 (0.12; 0.21)
**Alcohol intake, %**				
Low intake	0.94 (0.92; 0.96)	0.95 (0.92; 0.96)	0.92 (0.84; 0.96)	0.95 (0.92; 0.97)
High intake	0.05 (0.03; 0.08)	0.05 (0.03; 0.08)	0.08 (0.04; 0.16)	0.05 (0.03; 0.08)
**Smoking, %**				
Former	0.31 (0.27; 0.36)	0.24 (0.21; 0.26)	0.26 (0.18; 0.36)	0.23 (0.18; 0.28)
Current	0.28 (0.24; 0.32)	0.23 (0.20; 0.26)	0.26 (0.18; 0.37)	0.22 (0.17; 0.27)
Occasional smoker	0.08 (0.06; 0.11)	0.08 (0.06; 0.10)	0.09 (0.05; 0.17)	0.09 (0.06; 0.13)
No smoker	0.33 (0.29; 0.37)	0.45 (0.42; 0.48)	0.38 (0.29; 0.48)	0.46 (0.41; 0.52)

Data presented as mean for continuous variables or percentage for categorical variables, with their corresponding 95% CI. WC: waist circumference; PA: physical activity; MET: metabolic energy task; g: grams; min: minutes.

**Table 2:** Associations between travelling mode and adiposity markers.

	Cars	Public Transport	*p*	Cycling	*p*	Walking	*p*
Weight (kg)		β (95% CI)		β (95% CI)		β (95% CI)	
Model 1	1.00 (Ref.)	−7.33 (−9.00; −5.66)	<0.001	−5.40 (−8.35; −2.44)	<0.001	−8.60 (−10.5; −6.70)	<0.001
Model 2	1.00 (Ref.)	−4.88 (−6.53; −3.23)	<0.001	−6.06 (−8.96; −3.16)	<0.001	−6,29 (−8.18; −4.39)	<0.001
Model 3	1.00 (Ref.)	−4.78 (−6.43; −3.12)	<0.001	−6.02 (−8.88; −3.16)	<0.001	−6.20 (−8.09; −4.30)	<0.001
Model 4	1.00 (Ref.)	−4.77 (−6.42; −3.11)	<0.001	−6.03 (−8.90; −3.11)	<0.001	−6.16 (−8.05; −4.26)	<0.001
BMI (kg/m^2^)							
Model 1	1.00 (Ref.)	−0.91 (−1.45; −0.36)	<0.001	−1.88 (−2.98; −0.77)	<0.001	−1.56 (−2.21; −0.90)	<0.001
Model 2	1.00 (Ref.)	−1.20 (−1.75; −0.66)	<0.001	−1.70 (−2.73; −0.67)	<0.001	−2.02 (−2.66; −1.38)	<0.001
Model 3	1.00 (Ref.)	−1.19 (−1.73; −0.65)	<0.001	−1.69 (−2.71; −0.67)	<0.001	−2.00 (−2.64; −1.36)	<0.001
Model 4	1.00 (Ref.)	−1.18 (−1.73; −0.64)	<0.001	−1.70 (−2.72; −0.68)	<0.001	−1.98 (−2.63; −1.34)	<0.001
WC (cm)							
Model 1	1.00 (Ref.)	−4.71 (−6.12; −3.29)	<0.001	−4.99 (−7.66; −2.32)	<0.001	−5.35 (−6.95; −3.76)	<0.001
Model 2	1.00 (Ref.)	−3.52 (−4.87; −2.17)	<0.001	−4.73 (−7.18; −2.28)	<0.001	−4.71 (−6.22; −3.20)	<0.001
Model 3	1.00 (Ref.)	−3.47 (−4.81; −2.12)	<0.001	−4.69 (−7.11; −2.27)	<0.001	−4.66 (−6.17; −3.15)	<0.001
Model 4	1.00 (Ref.)	−3.46 (−4.80; −2.10)	<0.001	−4.71 (−7.13; −2.28)	<0.001	−4.62 (−6.13; −3.11)	<0.001

Data presented as β coefficient and their 95% confidence intervals. Car travellers were used as the reference group; therefore, the β indicate differences of each travel mode compared to cars travellers. All statistical analyses were incrementally adjusted by several confounding factors, including four models: Model 1 was unadjusted; Model 2 was adjusted by socio-demographic factors (age, sex, education and place of residency), Model 3 was additionally adjusted by lifestyle factors (smoking, alcohol, fruit and vegetable intake and sedentary behaviours) and finally, Model 4 was additionally adjusted by levels of leisure PA; WC: waist circumference; β: beta coefficient.

**Table 3:** Association between travelling mode and obesity.

	Car	Public Transport	*p*	Cycling	*p*	Walking	*p*
Overweight		OR (95% CI)		OR (95% CI)		OR (95% CI)	
Model 1	1.00 (Ref.)	0.56 (0.43; 0.74)	<0.001	0.61 (0.36; 0.86)	<0.001	0.56 (0.40; 0.77)	<0.001
Model 2	1.00 (Ref.)	0.57 (0.42; 0.76)	<0.001	0.62 (0.37; 0.87)	<0.001	0.54 (0.38; 0.75)	<0.001
Model 3	1.00 (Ref.)	0.58 (0.43; 0.78)	<0.001	0.63 (0.37; 0.87)	<0.001	0.55 (0.39; 0.77)	<0.001
Model 4	1.00 (Ref.)	0.58 (0.43; 0.78)	<0.001	0.63 (0.38; 0.89)	<0.001	0.55 (0.38; 0.77)	<0.001
Obesity							
Model 1	1.00 (Ref.)	0.56 (0.41; 0.76)	<0.001	0.51 (0.28; 0.93)	0.029	0.49 (0.33; 0.71)	<0.001
Model 2	1.00 (Ref.)	0.51 (0.37; 0.71)	<0.001	0.53 (0.30; 0.61)	0.031	0.41 (0.27; 0.61)	<0.001
Model 3	1.00 (Ref.)	0.51 (0.37; 0.72)	<0.001	0.55 (0.31; 0.98)	0.039	0.41 (0.28; 0.61)	<0.001
Model 4	1.00 (Ref.)	0.51 (0.37; 0.72)	<0.001	0.55 (0.31; 0.99)	0.048	0.41 (0.28; 0.61)	<0.001
Central obesity							
Model 1	1.00 (Ref.)	1.02 (0.82; 1.30)	0.829	0.51 (0.33; 0.81)	0.004	1.04 (0.78; 1.38)	0.801
Model 2	1.00 (Ref.)	0.66 (0.51; 0.86)	0.002	0.56 (0.35; 0.89)	0.014	0.64 (0.46; 0.88)	0.006
Model 3	1.00 (Ref.)	0.66 (0.51; 0.86)	0.002	0.56 (0.35; 0.89)	0.014	0.64 (0.47; 0.89)	0.007
Model 4	1.00 (Ref.)	0.66 (0.51; 0.86)	0.002	0.56 (0.35; 0.89)	0.014	0.64 (0.47; 0.89)	0.007

Data presented as odds ratio and their 95% confidence intervals. Car travellers were used as the reference group. All statistical analyses were incrementally adjusted by several confounding factors including four models: Model 1 was unadjusted; Model 2 was adjusted by socio-demographic factors (age, sex, education and place of residency), Model 3 was additionally adjusted by lifestyle factors (smoking, alcohol, fruit and vegetable intake and sedentary behaviours) and finally, Model 4 was additionally adjusted by levels of leisure PA. Overweight was defined as BMI ≥25.0 kg/m^2^; Obesity was defined as BMI ≥30.0 kg/m^2^; and Central obesity was defined as WC >88 and >102 cm for men and women, respectively.

## Data Statement

Data access is available on http://epi.minsal.cl/bases-de-datos/.

## Appendix A

ELHOC-RESEARCH Team members who contributed to this manuscript and therefore are co-authors: Solange Parra-Soto, Claire Richardson, Daniel Mondaca and Viktoria Baltnzi (Institute of Cardiovascular and Medical Sciences, University of Glasgow, Glasgow, United Kingdom); Alex Garrido-Méndez (Departamento de Ciencias del Deporte y Acondicionamiento Físico, Universidad Católica de la Santísima Concepción, Concepción, Chile); Nicole Lasserre (Escuela de Nutrición y Dietética. Facultad de Salud. Universidad Santo, Tomás. Chile); Fernando Rodriguez (Grupo IRyS, Escuela de Educación Física, Pontificia Universidad Católica de Valparaíso, Valparaíso, Chile); Cristian Álvarez (Laboratory of Human Performance. Quality of Life and Wellness Research Group. Department of Physical Activity Sciences. Universidad de Los Lagos. Osorno, Chile); Miquel Martorell (Centro de Vida Saludable, Universidad de Concepción, Concepción, Chile & Departamento de Nutrición y Dietética, Facultad de Farmacia, Universidad de Concepción, Concepción, Chile); Karina Ramírez and Ana Labraña (Departamento de Nutrición y Dietética, Facultad de Farmacia, Universidad de Concepción, Concepción, Chile); Natalia Ulloa (Centro de Vida Saludable, Universidad de Concepción, Concepción, Chile & Departamento de Bioquímica Clínica e Inmunología, Facultad de Farmacia, Universidad de Concepción, Concepcion, Chile); Gabriela Nazar (Departamento de Psicología y Centro de Vida Saludable, Facultad de Ciencias Sociales, Universidad de Concepción, Concepción, Chile); and Jaime Vásquez (Vicerrectoría de Investigación y Posgrado, Centro de Investigación en Estudios Avanzados del Maule (CIEAM), Universidad Católica del Maule, Talca, Chile).
